# Tactile-STAR: A Novel Tactile STimulator And Recorder System for Evaluating and Improving Tactile Perception

**DOI:** 10.3389/fnbot.2018.00012

**Published:** 2018-04-06

**Authors:** Giulia Ballardini, Giorgio Carlini, Psiche Giannoni, Robert A. Scheidt, Ilana Nisky, Maura Casadio

**Affiliations:** ^1^Department of Informatics, Bioengineering, Robotics and Systems Engineering, University of Genoa, Genoa, Italy; ^2^Marquette University and the Medical College of Wisconsin, Milwaukee, WI, United States; ^3^Feinberg School of Medicine, Northwestern University, Chicago, IL, United States; ^4^Division of Civil, Mechanical and Manufacturing Innovation, National Science Foundation, Alexandria, VA, United States; ^5^Department of Biomedical Engineering, Ben-Gurion University of the Negev, Beersheba, Israel; ^6^Zlotowski Center for Neuroscience, Ben-Gurion University of the Negev, Beersheba, Israel

**Keywords:** tactile stimulation, somatosensory function, skin stretch, skin brush, stroke, neurological disease, haptics

## Abstract

Many neurological diseases impair the motor and somatosensory systems. While several different technologies are used in clinical practice to assess and improve motor functions, somatosensation is evaluated subjectively with qualitative clinical scales. Treatment of somatosensory deficits has received limited attention. To bridge the gap between the assessment and training of motor vs. somatosensory abilities, we designed, developed, and tested a novel, low-cost, two-component (bimanual) mechatronic system targeting tactile somatosensation: the *Tactile-STAR*—a tactile stimulator and recorder. The stimulator is an actuated pantograph structure driven by two servomotors, with an end-effector covered by a rubber material that can apply two different types of skin stimulation: brush and stretch. The stimulator has a modular design, and can be used to test the tactile perception in different parts of the body such as the hand, arm, leg, big toe, etc. The recorder is a passive pantograph that can measure hand motion using two potentiometers. The recorder can serve multiple purposes: participants can move its handle to match the direction and amplitude of the tactile stimulator, or they can use it as a master manipulator to control the tactile stimulator as a slave. Our ultimate goal is to assess and affect tactile acuity and somatosensory deficits. To demonstrate the feasibility of our novel system, we tested the *Tactile-STAR* with 16 healthy individuals and with three stroke survivors using the skin-brush stimulation. We verified that the system enables the mapping of tactile perception on the hand in both populations. We also tested the extent to which 30 min of training in healthy individuals led to an improvement of tactile perception. The results provide a first demonstration of the ability of this new system to characterize tactile perception in healthy individuals, as well as a quantification of the magnitude and pattern of tactile impairment in a small cohort of stroke survivors. The finding that short-term training with *Tactile-STAR* can improve the acuity of tactile perception in healthy individuals suggests that *Tactile-STAR* may have utility as a therapeutic intervention for somatosensory deficits.

## Introduction

Many people with neurological diseases suffer from impairments of the motor and the somatosensory functions. Reliable methods to quantify somatosensory deficits are crucial for better understanding the pathophysiology of the diseases and for enhancing the detection of early symptoms and informing novel neuro-rehabilitative approaches to improve upper-limb functions and quality of life.

Impaired somatosensory function significantly affects the quality of daily living. Somatosensation is critical for autonomy in the environment and purposeful interaction with the external world. An example of a somatosensory ability of a healthy individual is identifying an object using only haptic perception, or stereognosis (Irving, 1968). It entails active haptic exploration (Jones and Lederman, 2006), and incorporates both movement control to manipulate the object with the fingers, and the sensory capacity to provide cues from texture, size, spatial properties, and temperature (Yekutiel et al., 1994). Other examples include perception of stiffness or other mechanical properties (Jones and Hunter, 1993; Leib et al., 2016), and sensing contact and friction forces for manipulation of objects and prevention of their slippage (Kandel et al., 2000; Johansson and Flanagan, 2009).

There are two main somatosensory systems that are vital to daily functions—kinesthetic and tactile. The kinesthetic system provides information about the position and movement of the body and limbs (proprioception) using muscle spindles and joint mechanoreceptors, and force information using the Golgi tendon organs (Winter et al., 2005; Proske and Gandevia, 2009, 2012). The tactile (or cutaneous) system provides information about contact with objects using mechanoreceptors in the skin (Demain et al., 2013). Information from these two systems is integrated in the central nervous system (Gurari and Okamura, 2014; Culbertson et al., 2018) together with predictions from internal representations (Körding and Wolpert, 2004) to create perception of the external world and the body schema, to plan and control movement (Morasso et al., 2015; Farajian et al., unpublished), and acquire skill (Vidoni et al., 2010). In this study we focus on the tactile system.

In the neurological assessment, somatosensory functions are most often subjectively assessed by clinicians using qualitative clinical scales (Winward et al., 1999; Scott and Dukelow, 2011). Several approaches are currently used to assess tactile acuity (Craig and Johnson, 2000), including: two-point threshold, gap detection (Stevens and Choo, 1996), and grating orientation. The latter is a reliable index of recovery following nerve damage (Van Boven and Johnson, 1994). An example of a quick and low-cost device to detect thresholds for mechanical stimuli is the Frey filaments (Von Frey, 1896; Johansson et al., 1980; Woolf, 1983; Lambert et al., 2009). However, all of these approaches evaluate static tactile acuity. By contrast, clinicians often assess somatosensation by touching the skin of the patients to evaluate their ability to detect the extent and the direction of a moving tactile stimulus. Quantifying such dynamic acuity during neurological examination remains difficult because of the limited sensitivity and reproducibility of the clinical tests.

The introduction of robotic technologies into clinical assessment and treatment has advanced the understanding and the treatment of motor functions in many neurological diseases (Prange et al., 2006; Kwakkel et al., 2008; Mehrholz et al., 2012; Norouzi-Gheidari et al., 2012; Basteris et al., 2014; Klamroth-Marganska et al., 2014). In contrast to this vast proliferation of robotic technologies in rehabilitation of motor functions, the somatosensory functions have received less attention. Specifically, robotics technology has been successfully used to quantify and characterize proprioceptive deficits in the research domain (Carey et al., 1996; Dukelow et al., 2010, 2012; Wilson et al., 2010; Simo et al., 2011; Semrau et al., 2013; Domingo and Lam, 2014; Aman et al., 2015; De Santis et al., 2015; Chisholm et al., 2016; Kuczynski et al., 2016; Maggioni et al., 2016; Marini et al., 2016, 2017), but their use in the clinical settings is still limited. One possible impeding factor in wider adoption of the several proposed technological solutions in the clinic is their high costs. To date, in this domain, the tactile system was almost neglected.

In comparison to the above-mentioned robotic technologies, tactile stimulation devices are often low cost, small, lightweight, and can be easily integrated into wearable technologies. These qualities make tactile stimulation technology attractive for rehabilitation and clinical assessment, especially in ambulatory conditions. Tactile feedback can be provided by using electrical and mechanical stimulations. Electrotactile stimulation involves passing an electrical current through the skin (Szeto and Saunders, 1982). It has been demonstrated that this type of stimulation has positive effects on motor performance, limb sensation, and the configuration of sensory evoked potentials of the paretic limb in people with chronic stroke (Peurala et al., 2002). Mechanical stimulation can be produced by vibration, pressure, or skin stretch (Demain et al., 2013; Culbertson et al., 2018). Specifically, vibrotactile stimulation is very prominent and simple to administer, and the frequency of the delivered vibration can be modulated to convey information (Sherrick et al., 1990). It has been shown useful, for example, to synthesize and deliver vibrotactile kinesthetic feedback to enhance stabilization and reaching actions performed with the arm and hand in neurotypical people (Krueger et al., 2017) and to improve proprioception (Cuppone et al., 2016). However, some users report continuous vibration to be annoying (Bark et al., 2008). Another limitation of the vibration approach is that the Pacinian corpuscles that detect vibration have large receptive fields, and therefore, the source of the vibration cannot be accurately localized (Bark et al., 2008).

In recent years, significant progress has been made in the development of devices for tactile stimulation that deform the skin by indentation or stretch (Drewing et al., 2005; Lévesque et al., 2005; Luk et al., 2006; Gleeson et al., 2010; Prattichizzo et al., 2012; Quek et al., 2014b, 2015a,b; Memeo and Brayda, 2016; Schorr and Okamura, 2017). There are many different mechanical approaches to applying skin stimulation, including a rotation of an end-effector on the skin (Bark et al., 2009; Chinello et al., 2016; Battaglia et al., 2017) or movement of a rigid end effector against the user’s fingerpad (Kuniyasu et al., 2012; Schorr et al., 2013; Quek et al., 2014a, 2015b). Skin stretch is very effective in providing the users with rich information; for example, stretch of the skin can augment perception of stiffness (Quek et al., 2014a), force magnitude (Paré et al., 2002), and friction (Provancher and Sylvester, 2009). Importantly, skin stretch can be used to convey directional information (Gleeson et al., 2009), and even replace kinesthetic information in navigation tasks (Guinan et al., 2013; Quek et al., 2014b, 2015a). A skin-stretch device was used to substitute for force in a teleoperated palpation, more effectively than the widely used vibration feedback (Schorr et al., 2015), and in a virtual peg-in-hole insertion task (Quek et al., 2015b). This task is often used for evaluation of robotic interfaces—participants have to insert an elongated peg into a narrow hole.

In most of these applications, skin stretch was applied in the fingertip (Pacchierotti et al., 2017) and it may be that in other locations with larger surface areas and more rough skin, it may be more effective to use brush stimulation. We define tactile brushing as a slight pressure while moving along the surface of the skin. Therefore, in the current work, we designed a device that can apply a stretch or a brush stimulation to different parts of the body, and focused on brush stimulation for our evaluation.

The long-term goal of our study is to develop a low-cost haptic device for assessing and rehabilitating somatosensation in subjects suffering from sensorimotor deficits. This device shall be able to apply skin-brush and skin-stretch stimuli to various parts of the body. Toward this goal, here we aimed at: (1) designing a first prototype of the device: the *Tactile-STAR*—a tactile stimulator and recorder, (2) validating its utility in the assessment and training of tactile acuity by collecting normative performance and training data in healthy human participants, and (3) demonstrating its ability to detect and quantify somatosensory deficits in a small cohort of stroke survivors.

## Materials and Methods

### System Design and Implementation

**FIGURE 1:**
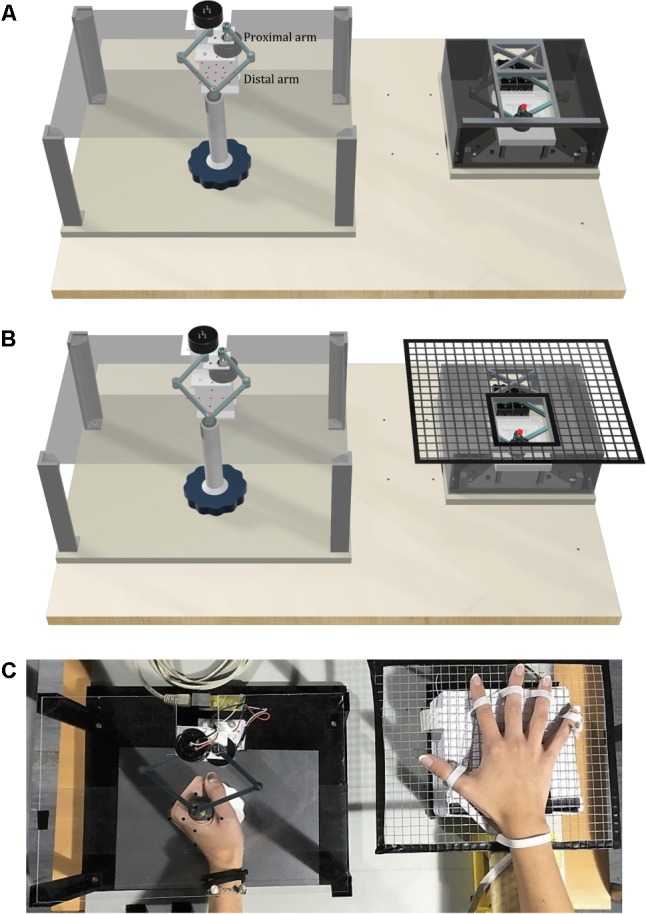
The *Tactile-STAR* system is composed of the recorder device (left) and the stimulator device (right). **(A)** The basic configuration of the *Tactile-STAR* that was validated in a test with healthy individuals in Experiment 1. Red targets used in the verification study involving healthy participants are shown projected onto the transparent plane situated above the recorder’s handle. **(B)** The modified configuration validated with stroke survivors in Experiment 2. A rigid mesh support grid was added to the stimulator on the right, and the targets (left) were modified such that the participants only had to match stimuli in the cardinal directions. **(C)** Picture of the device used by a healthy control subject in Experiment 2. In the text we use the word “distal” for referring to the links distal from the motors or the potentiometers, i.e., close to the end effector, and the word “proximal” for the links close to the motors or the potentiometers.

The *Tactile-STAR* system is composed of two interconnected devices (**Figure 1**). The first device, the *stimulator*, is an actuated pantograph structure driven by two servomotors. The end-effector of the stimulator is covered by a cap of rubber material that moves in contact with the skin. Depending on its mechanical configuration, the device can provide different forms of tactile stimulation (see below). The second device, the *recorder*, is a passive pantograph that measures the motion of its handle (its end effector) using two precision potentiometers. Both systems interface to an Arduino microcontroller system, which also interfaces to a laptop computer that runs a LabVIEW^®^ 2016 “virtual instrument” (National Instruments Inc.) that monitors the state of both systems, controls the state of the stimulator device, and provides user interfaces for the experimenter and the research participant.

#### The Pantograph Structures

**FIGURE 2:**
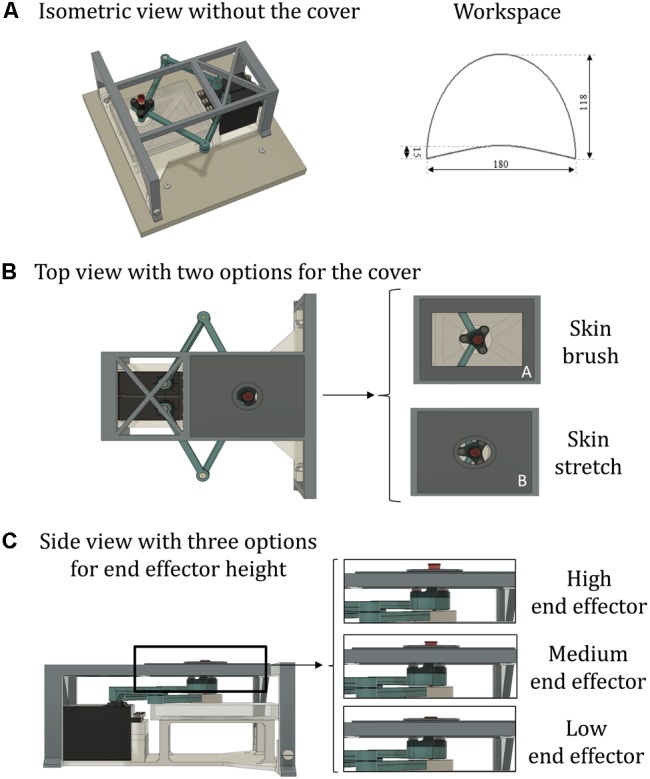
The stimulator device. **(A)** An isometric view without the cover (left), and the workspace of the device (right). **(B)** Top view with different options for the cover. The figure on the left shows the circular aperture (7 mm radius) for skin stretch; on the right is shown: A—rectangular aperture (60 mm × 40 mm) for skin brush; B—elliptical aperture (22 mm × 18 mm) for skin stretch. The center of each aperture is centered on the center of the workspace. The aperture on panel **(A)** is the one used for both validation experiments presented in this report. **(C)** Side view with three options for end-effector height. For the skin-stretch stimulation, the tip of the end-effector is raised from 1.5 to 2.5 mm (medium and high end-effector configurations) above the surface upon which the tested body part rests. For skin-brush stimuli, the tip of the end-effector is only slightly raised above the surface where the limb rests (<1.5 mm, low end-effector configuration).

Both the stimulator and the recorder have identical pantograph structures with four links and two degrees of freedom (Campion et al., 2005); see section 1. “Direct and Inverse Kinematics of the Stimulator and Recorder Devices” (Supplementary Figure S1) of the Supplementary Material for forward and inverse kinematics. The current prototype (**Figure 1**) has a symmetric design such that the left and right links of the device are identical, with lengths of 5.75 cm for the proximal links and 6.75 cm for the distal links. We selected these dimensions to obtain a workspace large enough to stimulate almost half of the lower arm length, which ranges between 24.34 cm for females and 26.99 cm for males (Gordon et al., 1989; **Figure 2B**, B). The mechanical linkage was required to be rigid and lightweight. The rigidity is important because the linkage must maintain its shape and not bend when stimulating the skin. To increase rigidity without adding weight, we designed the links with a T-shaped cross-section (see Supplementary Figure S2). The arm links were connected with a ball-bearing (MinebeaMitsumi Inc.) fixed into one link, and a metal axle rigidly connected to the adjoining link. We fixed a plastic ring on the top of the axle in order to maintain the axle in the correct perpendicular orientation during all movements. By configuring the connection between the two arms in this way, we ensured that: (1) the links were on two different levels to prevent collisions between the arms; and (2) the resulting workspace was maximized for the given link dimensions, and (3) there were no unreachable points inside the workspace. All the parts of the pantograph structure were manufactured by a Form 2 stereolithographic printer (FormLabs Inc.), with a resolution of 0.05 mm (see section 2. “Development of the Device Through 3D Printers” in Supplementary Material for more details).

#### The Stimulator

The arms of the pantograph structure are connected on one side to two servomotors (Parallax Standard Servo, Parallax Inc.) and on the other side to the end effector (**Figure 2B**, A). Each servomotor has a range of motion of 180°. To ensure against sliding between the proximal link and the motor, a linchpin is used to lock the link to the motor. Although the selected servomotor does not normally provide an output signal corresponding to its angle of rotation, it is possible to measure that signal by tapping into the servo’s internal potentiometer to derive a voltage that is proportional to the angle of rotation. We read that signal to verify that each commanded position was reached correctly. The end-effector is placed on top of the upper pantograph link distal form the motor and it is composed by a base layer with a hollow cylinder. In the cylinder, there is a fillet expansion insert that houses a screw. The head of the screw is the tip of the end effector that would be in contact with the skin. To make the sensation more comfortable while increasing the friction, it is covered by a cap of rubber material (IBM ThinkPad TrackPoint Cap). This screw allows regulating the height of the tip of the end effector, thus providing different tactile sensations (**Figure 2C**).

To have a skin-stretch sensation, it is necessary to place over the stimulator device another structure with an aperture within which the end effector moves. The design of this structure is modular, such that it is possible to use different sizes and shapes of the aperture and the end-effector, without changing the entire structure (**Figure 2C**). Therefore, the sensation created by the tactile stimulator can range from light-touch to skin stretch, depending on the shape of the end-effector and on the size of the aperture. The aperture structure placed over the pantograph also serves as a support by sustaining weight placed on it by the user’s arm. This structure is rigidly connected to a base-platform, upon which the motors that move the robot arms are fixed. To ensure that the end-effector remains at all times perpendicular to the horizontal plane without bending, the base platform also has a plastic plane that supports the distal, lower link of the pantograph, immediately below the end-effector. To decrease friction during sliding, the lower link’s contact point is covered with a 2-mm layer of polytetrafluoroethylene (PTFE). When the device is operated and the end-effector touches the skin, this contact causes friction. Therefore, in each trial, we recorded the reading of the potentiometers, and monitored whether or not the end-effector motion was affected by the friction. During experimental setup, we adjusted the height of the end-effector such that the tactor did not become stuck at any time, and that it would arrive to all desired targets.

#### The Recorder

The proximal links of the pantograph structure are connected to two rotational, single-turn potentiometers (Vishay 132, Vishay Intertechnology, Inc.) that have a linear taper, a resistance of 2 KΩ ± 3% and a linearity of ±0.5%. The distal links are connected to a handle as described below (**Figure 1**). The recorder has a baseplate structure designed such that the centers of rotation of the two potentiometers are positioned relative to one another in an identical manner as the servo motor centers of rotation on the stimulator device. Thus, the pantograph structure of the recorder is exactly the same as that of the stimulator. The lower distal arm is connected through a brass axle to the handle of the device. The handle itself is composed of two parts: (1) a cylinder (1 cm radius × 10 cm high), which is intended to be held in the participant’s hand, and (2) a low-friction disk that supports the hand’s weight. The bottom surface of the disk is coated with PTFE to decrease friction as it slides over the top surface of the rigid baseplate. The recorder can serve two purposes: (1) in its *passive mode*, the user can move the recorder’s handle to match the direction and amplitude of the tactile stimulus generated by the stimulator, or (2) in the *active mode*, the user can move the handle as a master manipulator to control the tactile stimulator as a slave.

The stimulator and recorder are each mechanically connected to a larger rigid ground plane (**Figure 1**). The two devices can be mounted to the ground plane in several different configurations and in this way, we can stimulate either the right or the left hand and use the handle with the opposite hand. The distance between the two devices can be modified according to individual participant anthropometric measurements.

#### System Control Architecture (**Figure 3**)

**FIGURE 3:**
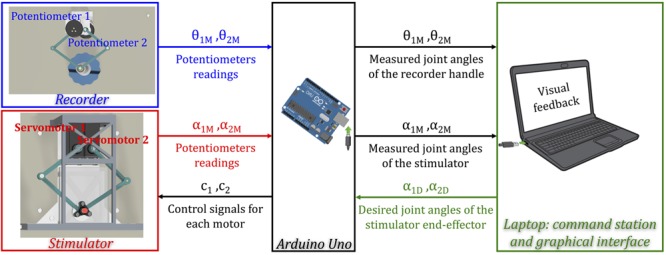
A schematic representation of the control scheme of the *Tactile-STAR*. An Arduino Uno collects data from the potentiometers integrated into the two devices, and sends the commands to the stimulator servomotors. The laptop receives from the Arduino measurements of the angles from both the recorder and the stimulator, and sends the desired angles to the stimulator. The laptop provides both the graphical interface for the experimenter and online feedback to the participants during the experiment.

A circuit board based on the Atmel ATmega328p microcontroller (Atmel Inc.) performs analog-to-digital conversion on four input voltage signals derived from the two potentiometers embedded within each device. An additional analog input is reserved for a force sensor that can be inserted optionally inside the stimulator device to measure the force applied by the end-effector to the skin. The microcontroller sends as outputs an independent control signal for each of the two motors of the stimulator. These two Pulse-Width-Modulation (PWM) signals set reference angular positions for the two motors, which enforce those positions under internal, closed-loop, feedback control. The microcontroller also relays the potentiometers signals from the stimulator and the recorder to a laptop computer, and receives as input from the laptop the desired angular positions of the stimulator joints (see Supplementary Figure S3 for more details on electrical connections). The laptop runs a program that controls the system, provides visual feedback of the task to the research participant, and provides a user interface for the experimenter.

The *Tactile-STAR* system can work in two distinct modes. In the *passive mode*, the user moves the handle of the recorder to match the direction and amplitude of motions produced by the tactile stimulator. The laptop computes the desired joint angles of the stimulator from the desired end-effector path using the kinematic equations reported in section 1. “Direct and Inverse Kinematics of the Stimulator and Recorder Devices” in Supplementary Material. The joint angles from the stimulator’s potentiometers are recorded to verify that the target positions commanded by the laptop and controller are reached correctly. The joint angles of the recorder are measured with its potentiometers to verify that the participant correctly replicates the stimulation. In the *Tactile-STAR’s active mode*, the user can move the handle of the recorder as a master manipulator to teleoperate the tactile stimulator as a slave. In this mode, the joint angles of recorder device are used to set the desired joint angles for the stimulator. In both modes, scale factors may be programmed between the workspaces of the two devices in order to break the nominal 1:1 correspondence between the recorder’s handle and the stimulator’s end-effector.

#### Stimuli

The Tactile-STAR stimulator can produce two distinct forms of tactile stimuli: skin-brush and skin-stretch stimulation. As for the skin-stretch stimulation, the tip of the end-effector is raised from 1.5 to 2.5 mm (**Figure 2C**) above the surface upon which the tested limb (or body part) is resting and moves inside a smaller aperture (elliptical shape: 0.022 m × 0.018 m) with raised margins. As for the skin-brush stimulation, the aperture is larger (rectangular shape: 0.060 m × 0.040 m), its margins are at the same level of the surface where the limb is resting, while the tip of the end-effector is slightly raised above it (<1.5 mm; **Figure 2B**).

#### Software

We used a custom LabVIEW 2016 program, along with the LabVIEW Interface for Arduino (LIFA), to control the stimulator and recorder devices, to provide real-time visual feedback to the research participant, and to provide an experimental control interface for the experimenter. The custom LabVIEW program allows the experimenter to define experimental task parameters, including participant anthropometrics. The program also stores position (and optionally force) data to disk for subsequent (offline) analysis.

### Technical Validation

**FIGURE 4:**
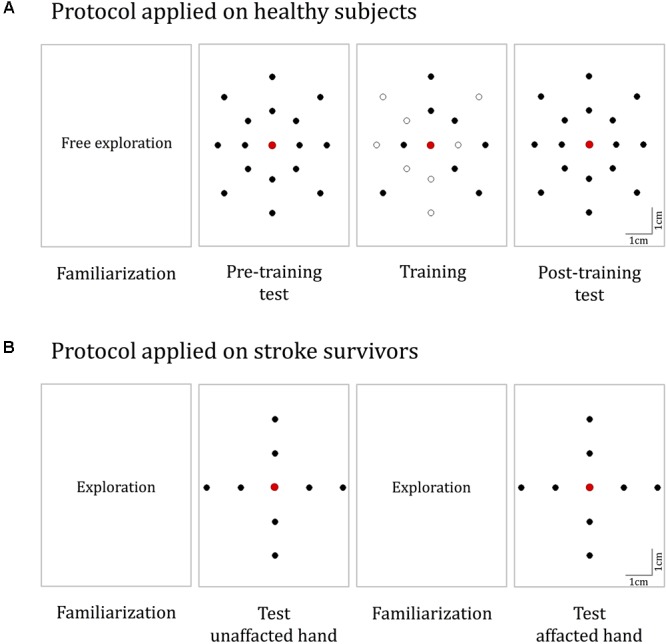
Experimental protocols. **(A)** The protocol for healthy participants. It consisted of four phases: familiarization, pre-training test, training, and post-training test. During familiarization, the *Tactile-STAR* was placed in active mode and the subjects moved the handle of the recorder device to produce stimulator end-effector motions that were identical in magnitude and direction to the movements of the recorder endpoint (handle). In both the test phases, 16 test targets located on two concentric ellipses were presented five times in random order. During training, a subset of eight targets was presented nine times each. **(B)** The protocol for the validation experiments with stroke survivors. The test was performed on both hands, and for each hand, we had both a familiarization and a test phase. The targets for the test phase are placed in the four cardinal directions on two concentric circles.

We validated the accuracy and precision of the stimulator’s control of end-effector position using an optical motion tracking system. Three infrared cameras (V120 slim, NaturalPoint Inc., OR, United States; software: C++ custom modification of NaturalPoint SDK) recorded the three-dimensional position of an active infrared marker that we fixed to the top of the end-effector. We defined 24 spatial targets that were distributed across four elliptic arcs that spanned the stimulator’s entire workspace (**Figure 2A**, right panel; **Figure 4**). We programmed the stimulator to reach each of the targets 10 times, and to stay in the commanded position for 1 s. For each target point, the constant error was less than 0.035 mm (mean ± SD 0.002 ± 0.018 mm), while the variable error was less than 0.005 mm (mean ± SD 0.002 ± 0.001 mm).

We repeated the same calibration procedure for the recorder. We manually positioned the tip of the end-effector on the same target points used for calibrating the stimulator, and verified that we reached the correct positions using the user interface of the recorder device. We then recorded these positions using both the encoders of the recorder and the optical system. For each target point, the constant error was less than 0.009 mm (mean ± SD 0.001 ± 0.004 mm), while the variable error was less than 0.009 mm (mean ± SD 0.002 ± 0.001 mm). Thus, the errors obtained with this low-cost prototype were negligible in the experimental settings used for the validation testing described below.

### Verification Study Involving Human Participants

All participants provided written informed consent to participate in the study procedures, which were approved by a local institutional ethics committee—Comitato Etico ASL3 Genovese (Italy)—in compliance with the Declaration of Helsinki.

### Verification Study Involving Healthy Participants

We sought to perform a first functional test of the *Tactile-STAR* system with young participants without somatosensory deficits to verify its ability to characterize and affect tactile perception. Participants were tested before and after 30 min of perceptual training (described below) using the *Tactile-STAR* device. We tested two main hypotheses: (1) the ability to identify correctly distinct skin-brush stimuli applied to the palm of the hand is not uniformly distributed across the palm; (2) the ability to correctly identify distinct skin-brush stimuli applied to the palm of the hand can improve following a short period (∼30 min) of practice.

#### Participants

Sixteen healthy young right-handed participants (eight females, 24 ± 2 years) participated in a single-session experiment wherein they interacted with the *Tactile-STAR* for approximately 45 min. All participants were naïve with respect to both the device and the experimental procedures.

#### Experimental Set-Up

Participants sat on a chair in front of a table upon which we placed the *Tactile-STAR* system. The *recorder* device was centered on the participants’ midline, and the *stimulator* device was placed on their right side (**Figure 1C**). Prior to testing, the *stimulator* was configured to stimulate the palm of the hand with a low end-effector profile. To prevent fatigue, the right arm was supported against gravity by a fixture placed next to the chair. The *stimulator* device had the center of its workspace aligned with the center of the right-hand palm. To prevent visual feedback of the stimulator’s position and motion, we added an opaque box over the tactile stimulator, thus hiding the mechanical structure from view. We also added a transparent plane on top of the recorder device where we projected visual targets (red dots; 1.5 mm radius; **Figures 1A,B**) that the stimulator could reach during testing. During the experiment, the participants did not use headphones. However, they reported that the background noise was higher than the device noise and that they relied on their somatosensation and not on acoustic feedback for solving the task.

#### Protocol

During testing (i.e., phase 2 and phase 4 of the experimental protocol; see below), the Tactile-STAR produced 16 unique tactile skin-brush stimuli of varying amplitudes and directions relative to the center of the stimulator’s workspace (and thus, relative to the center of the palm; **Figure 4A**). The stimulator’s end-effector, in light contact with the skin, made movements from the center of the workspace outward to targets placed on two concentric ellipses, resulting in center-out brushing stimulation on the participant’s palm. The dimensions of the axes of the inner ellipse were half of the respective axes of the outer ellipse (outer ellipse axes: 4 and 5 cm). The larger axis was aligned along the proximal–distal direction while the minor axis was aligned along the medio-lateral direction. Eight targets were equally distributed (45° apart) on each ellipse.

The experimental protocol consisted of four sequential phases (**Figure 4A**):

##### Phase 1: familiarization

The purpose of this phase was to allow participants to gain familiarity with the spatiotemporal characteristics of skin-brush stimulation. The *Tactile-STAR* was placed in active mode and participants used the recorder’s handle to freely explore the stimulator’s end-effector workspace. When the participants moved the handle of the recorder device, the stimulator device produced an end-effector motion that was identical in magnitude and direction to the movement they made. This phase continued for a minimum of 2 min and a maximum of 4 min.

##### Phase 2: pre-training test

The purpose of this phase was to assess each participant’s ability to discriminate between skin-brush stimuli of different magnitudes and directions (see section “Protocol”), and to use those stimuli to guide the planning and execution of goal-directed reaching movements. To do this, the *Tactile-STAR* was placed in passive mode and the tactile stimulator presented skin-brush stimulation to the palm of the hand using end-effector trajectories that moved from the central position to one of the target positions at a constant speed of 0.02 m/s. Upon reaching the target, the end-effector held its position as the participants moved the handle of the recorder device with their non-dominant hand until they believed that they had reached the corresponding target. Then, they held this position for a minimum of 0.5 s and declared to the experimenter that they had identified the stimulus. After having done so, they were instructed to return the handle of the recorder to the central position, and the stimulator returned to the start position at the maximal speed of the motors. After a pause of 1.5 s, the next stimulation trial started. Each of the 16 test targets was presented to the participant five times in random order (80 trials total). Participants received no feedback about their performance either during or after training.

##### Phase 3: training

The purpose of the training phase was to provide participants with extended practice in a stimulus-discrimination and replication task designed to encourage sensorimotor learning of the mapping between the motion of the recorder device’s handle and the motion of the stimulator’s end-effector. Each trial in the training phase had two parts. First, as in phase 2, participants were presented with tactile skin-brush stimulation as the end effector moved at 0.02 m/s from the central target to each of eight training targets selected from the set of 16 testing targets (**Figure 4A**, training). When the end-effector arrived at the target, that position was held for 1.5 s before returning at maximum speed to the central position. Second, the participant had to replicate with the non-dominant hand the handle motion corresponding to the skin-brush stimulation they had just experienced. To encourage sensorimotor learning in this training phase, the Tactile-STAR was placed in active mode during movement replication such that the participants received tactile feedback corresponding to motions they made during the replication trials; i.e., the stimulator replicated the motion of the recorder. In other words, participants received state feedback in the stimulated hand that corresponded to the position and motion of the recording hand. When the participant believed that they had arrived at the cued target, they declared that fact to the experimenter and then returned the handle to the central “home” position. If they had erred and reached the wrong target, they would hear an audible, non-startling error tone, and the same stimulus was repeated until the participants correctly interpreted it. Inter-trial intervals were nominally 1.5 s.

During training, participants performed three “training sets” that were separated by 3-min pauses to minimize to likelihood that participants might experience fatigue. In each training set, each of the eight training stimuli was presented three times in pseudo-random order, with the constraint that the same stimulus could not be presented more than two times in a row. To evaluate the learning without spatial accuracy biases that can arise due to the inertial anisotropy of the arm and hand (Gordon et al., 1994; Simo et al., 2011), or due to differences in the sensitivity to the stimulation, the same training target pattern was rotated 45° such that there were eight possible target configurations (one for every two participants). Across the participant group, each of the 16 targets was included in the training set of eight participants.

##### Phase 4: post-training test

The protocol in the post-training test phase was identical to that in the pre-training test phase (i.e., phase 2).

#### Data Analysis

We defined *final hand position* as the recorder’s handle location at the moment the participant declared he/she had arrived at the desired target. We defined the *final target* as the target with the smallest Euclidean distance from the final hand position. When the participants moved the handle of the recorder device, they were instructed to choose one of the 16 possible targets displayed on the transparent plane on top of the recorder device. Thus, we used the minimal Euclidian distance to identify which one of these 16 targets the participant indicated as correspondent to the perceived stimuli. Our primary outcome measure was the percentage of stimuli correctly perceived and replicated by the user (i.e., *percentage of correct responses*).

We used the Kolmogorov–Smirnov test to assess normality of the data distribution. For all data sets, the null hypothesis that these data come from a standard normal distribution was rejected at the 5% significance level. We expected this result, because the metrics we chose describe the percentage of targets recognized correctly. The percentage (unless well in the middle of the range) is expected to be distributed binomially, and violate the assumption of normality. Therefore, we used non-parametric tests that are based on rank statistics for testing our hypotheses.

Specifically, to test our first hypothesis (i.e., that the ability to correctly identify distinct skin-brush stimuli is not uniformly distributed across the palm), we applied the Friedman test to the *percentage of correct responses* obtained by each participant for each stimulus during both experimental test phases. To confirm the results obtained with the primary outcome in the test sets, we repeated the same analysis comparing the first and last training sets.

To test our second hypothesis (i.e., that the ability to correctly identify distinct skin-brush stimuli applied to the palm of the hand can improve following a short period of practice), we used the Wilcoxon signed-rank test to compare the percentage of stimuli correctly perceived in the pre- and post-training test phases. We also evaluated the *number of attempts* participants made before correctly interpreting each stimulus during the training phase.

Then, to identify which aspects of target acquisition were affected by the tactile stimulation, and test whether the potential benefits of training were specific to the trained targets or generalized to untrained targets, we performed follow-up analyses. The purpose of these exploratory investigations was to gain a preliminary understanding of what may be the strengths and weaknesses of our novel stimulation device and training protocol, and therefore, in these follow-up analyses, we did not correct for multiple comparisons. Another reason for this decision was that our follow-up tests were not independent, and the probability of making at least one Type I error would then be less than Bonferroni or Holm–Bonferroni assume. However, we also verified and report whether our results were robust against Holm–Bonferroni corrections.

We computed the following additional metrics:

##### Correct direction (%)

Percentage of stimuli in which the participants correctly interpreted the direction of the stimulation, independent from the perception of the amplitude. We inferred that the direction was identified correctly if the target that was indicated by the participant was in the same direction of the correct one.

##### Correct amplitude (%)

Percentage of stimuli in which the participants correctly interpreted the amplitude of the stimulation, independent from the perception of the direction.

We calculated these metrics for all the targets, and also separately for (a) the trained and untrained targets and (b) the targets of the outer and the inner ellipses.

Finally, we computed:

##### Nearest targets (%)

To compute this metric, we considered the answer correct if the participant indicated as perceived stimulus the correct target or one of its three nearest neighbors. This metric would be higher than the percentage of correct answers if the errors were due to insufficient perceptual resolution. Two of the nearest neighbors have the same amplitude as the correct target, and the third has the same direction.

To confirm the results obtained in the test phases, we repeated the same analysis for the training block by comparing the first and the last trial set. The threshold of statistical significance was set at *p* = 0.05.

### Validation Study With Stroke Survivors

We sought to provide a first proof-of-concept demonstration that the *Tactile-STAR* system is able to detect deficits of tactile perception in participants with neurological diseases. We hypothesized that the device would be able to identify significant stroke-related differences in tactile perception between the two hands, and that these differences would not be observable in healthy controls.

#### Participants

Three chronic stroke survivors (two females) participated in the experiment, as did three healthy controls matched for gender and age (±2 years). Each participant was enrolled by a neurologist and a physiotherapist, who performed the clinical evaluation (**Table 1**).

**Table 1:** Data of the stroke survivors.

Subject data													
	Age range (years)		E	PH	DD (years)		Lesion location					
P1	40–45		I	R	12		Left basal ganglia, internal capsule, and parietal lobe					
P2	66–70		H	L	2		Right thalamus					
P3	66–70		H	L	12		Right fronto-parietal pre-Rolandic					
**Clinical test scores**													
	**FMA**			**MAS**				**NAS**		**Vibration**			
	**A–D**	**H**		**Wrist**		**Fingers**	**Thumb**	**P**	**S**	**Left**		**Right**	
	**0–66**	**0–12**		**0–4**		**0–4**	**0–4**	**0–3**	**0–2**	**Elbow**	**Wrist**	**Elbow**	**Wrist**

P1	22	10		2		2	2	3	1	8	7	8	7
P2	26	9		3		2	1	3	1	4.5	4.5	6.5	6.5
P3	17	2		1		1	1	2	0	6	5.5	4.5	6

*Top rows: age, paretic hand (PH; L, left; R, right), the etiology (E) of ictus: ischemic (I)/hemorrhagic (H), the disease duration (DD) in years, and the location of the lesion. Bottom rows: clinical tests scores. FMA, Fugl–Meyer Assessment; MAS, Modified Ashworth Scale (Bohannon and Smith, 1987); NAS, Nottingham Assessment Scale (P, proprioception; S, stereognosis); vibration tested with the tuning fork.*

#### Experimental Set-Up

The experimental set-up described above was adapted for use by participants with a neurological injury. Since many stroke survivors have difficulty keeping the fingers of their affected hand extended, we added a wire grid (with 1 cm spaces between the bars) to the box over the stimulator. The central part of the grid was open in correspondence with the aperture of the stimulator device so as not to interfere with the end-effector motion (**Figures 1B,C**). An elastic band, adjustable in size and position for each participant, was used to keep the fingers comfortably opened and to hold the wrist on top of the grid (**Figure 1C**). When positioned correctly, the center of the palm corresponded to the center of the stimulator’s workspace. The position of the participant with stroke was the same as for the healthy participants when the right hand was tested. When we tested the left hand, the stimulator was positioned under the left hand, and the recorder was in front of the participant.

#### Experimental Protocol

We simplified the protocol with respect to the previous task in terms of the number and spatial distribution of the stimuli (**Figure 4B**). Here, we presented eight stimuli that tested two different amplitudes (5 and 2.5 cm) along the four cardinal directions. Since we expected that stroke survivors might have difficulty moving the matching device with the impaired hand when the unimpaired hand was tested with the stimulator device, we asked the participant to indicate verbally the target corresponding to the perceived stimulus. Both hands were tested, and the protocol was identical for the two hands. We did not test training effects in this protocol. The order in which the two hands were tested was the same for the stroke survivor and the related control participant—we first tested the right hand, and then the left.

Before each test, there was a familiarization phase in which the experimenter moved the handle of the matching device controlling the tactile stimulator motion. In this phase, participants familiarized themselves with the perception of tactile stimuli across all of the workspace, and specifically with stimuli having the same amplitude and directions as the ones used in the test phases.

In the two test blocks, each stimulus was presented five times in a random order, with no more than three consecutive repetitions of a same stimulus. When the end-effector reached the target position, the participant had to indicate the perceived stimulus. After the tactile stimulator returned to the central position, if a participant was not able to identify the stimuli, he/she could ask to repeat the stimulation up to three times. The successive stimulation started after a pause of 1.5 s. Participants did not receive any feedback about their performance. The experiment lasted about 30 min. Participants were free to stop the experiment at any time if they were tired or needed a break.

#### Data Analysis

We followed a single-subject design, and tested the differences in tactile acuity between the right and the left hand within each participant by using the Wilcoxon signed-rank test. Our primary performance measure was the “*percentage of correct responses*” and we decomposed this metrics by looking at the percentage of correct responses referred either to the correct identification of direction or amplitude of the stimuli (see section “Validation on Healthy Participants”). The stimuli were ordered taking into account the symmetry between the two hands (i.e., by mirroring the targets on the left hand to make them corresponding to the same on the right hand). Threshold for significance was set at *p* = 0.05.

## Results

### Validation on Healthy Participants

The tactile sensibility of 16 healthy participants was tested before and after 30 min of training. We tested two main hypotheses. Hypothesis 1 proposed that the ability to correctly identify distinct skin-brush stimuli applied by the device would not be uniformly distributed across the palm of the hand (i.e., that there would be a significant difference in perceiving brushing stimuli moving in different directions and of different extents relative to the center of the palm). Hypothesis 2 proposed that the ability to correctly identify distinct skin-brush stimuli applied to the palm can improve following a short (∼30 min) period of practice. We tested the two hypotheses in the experimental test sets and then we verified that the data from the training set confirmed results obtained in the test sets.

### Test Block Performance

**FIGURE 5:**
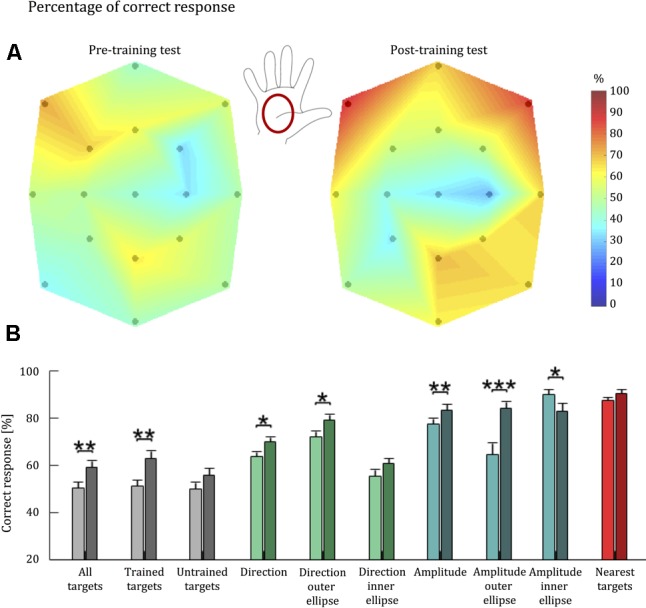
Tactile acuity of healthy individuals before and after training with the *Tactile-STAR*. **(A)** A colormap of the percentage correct responses for brush stimuli as a function of palm location. 100% corresponds to red, while 0% corresponds to dark blue. The black dots indicate the coordinates of the targets reached by the stimulator device, which started moving from the central target. The colors associated to intermediate coordinates were obtained by linear interpolation from the test points. Between the two colormaps, the illustration of the right hand shows where the stimuli were applied. The central position of the map corresponds to the center of the palm. **(B)** Bars represent population average percentage correct responses for each parameter: correct target (gray), correct direction (green), correct amplitude (blue), and nearest targets (red). Light colors are associated with performance before training; dark colors are associated with performance after training. Error bars indicate the standard error of the mean. ^∗^*p* < 0.05, ^∗∗^*p* < 0.01, ^∗∗∗^*p* < 0.001.

We visualized each participant’s ability to discriminate tactile stimuli (Hypothesis 1) by presenting, for each target, a colormap corresponding to the percentage of trials in which the user correctly identified the corresponding stimulus (**Figure 5A**). Colors for intermediate points were obtained via linear interpolation. A Friedman test detected a significant difference in the identification of the stimuli associated to different target locations both in the pre-training test (*p* < 0.001) and the post-training test blocks (*p* < 0.001). To test Hypothesis 2, we compared stimulus replication accuracy in the post-training test block to performance in the pre-training test block (**Figure 5B**).

Overall, we found a significant improvement for all the targets (*p* = 0.004), and for the trained (*p* = 0.004), while for the non-trained targets (*p* = 0.051) we did not reach the threshold of significance; that is, about 30 min of training led to an improvement for the trained targets, whereas improvement was not significant for the untrained targets. Analysis of individual participant’s performance revealed that the significant group effects were driven by 15 of the 16 participants, who improved their performance in the trained targets. By contrast, the lack of a significant effect for untrained targets was driven by four participants: whereas 12 of 16 subjects improved their performance at the untrained targets pre-to-post training, performance decreased slightly for three participants, and one participant did not change his performance pre-to-post training.

#### Detection of Stimulus Direction

To further understand the effects of short-term training with the *Tactile-STAR*, we repeated the analysis considering only the ability to correctly identify stimulus direction. Here, we considered a “correct answer” one that discriminates the direction of a stimulus independently from its amplitude. Across all targets, the Wilcoxon signed-rank test identified a significant improvement in the detection of stimulus direction for all the targets (*p* = 0.015), although this improvement was driven mainly by trials involving the trained targets (*p* = 0.015) and stimuli corresponding to targets on the outer ellipse (*p* = 0.017). Stimuli corresponding to untrained targets and to stimuli corresponding to targets on the inner ellipse did not reach statistical significance when analyzed separately (*p* > 0.05).

#### Detection of Stimulus Amplitude

We also isolated the ability to correctly identify the amplitude of stimuli by considering as “correct” those responses that replicated stimulus amplitude (i.e., short vs. long) regardless of movement direction. The Wilcoxon signed-rank test identified a significant improvement in the detection of all targets (*p* = 0.007), as well as the trained (*p* = 0.007), but not for the untrained targets (*p* = 0.087). We also find an improvement for the larger (*p* < 0.001) and the shorter stimuli (*p* = 0.041).

#### Nearest Neighbor Analysis

For this analysis, we considered a given response as “correct” if the participant’s response indicated one of the cued targets’ three nearest targets. Two of the nearest targets have the same amplitude as the cued target, while the third has the same direction. The value of the nearest neighbor parameter was always over 70%, indicating that even if the subject did not match the correct target identically, in most cases the error did not exceed one target distance. Participants had the same high level of performance both for trained and untrained stimuli. No training-dependent improvements were observed for this parameter regardless of how we subdivided the stimuli (*p* ≥ 0.124). Thus, improvements observed with other indicators were mainly due to improvements in the resolution of stimulus recognition.

The significance obtained for the two main hypotheses was robust against Holm–Bonferroni corrections. In contrast, most of the significant effects in our follow-up analysis in the test and training data sets would not survive these corrections. Therefore, testing more subjects will be necessary to fully understand which aspects of the tactile stimulation influence the performance improvements.

### Training Block Performance

**FIGURE 6:**
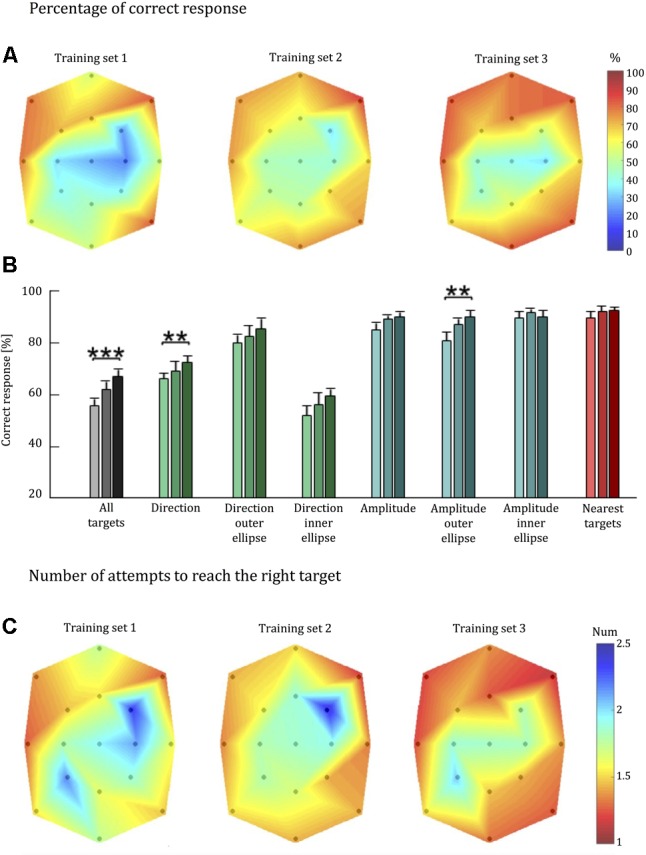
Tactile acuity of healthy individuals during training with the *Tactile-STAR*. **(A)** Colormap of the percentage correct responses as in **Figure 5**. **(B)** Population average percentage correct responses as in **Figure 5B**. **(C)** Colormap of the number of attempts needed to correctly identify the stimulus. Colors closer to blue indicate a larger number of wrong attempts to identify the stimulus. ^∗∗^*p* < 0.01, ^∗∗∗^*p* < 0.001.

We analyzed training set data as an independent challenge of our two hypotheses. First, we considered the percentage of “correct answers” considering only the initial answers given by each subject. Next, we took into account the number of attempts needed to yield a correct response. The performance indicators were computed for cued targets in the first and last training blocks. Friedman test of Hypothesis 1 detected a significant difference in the identification of the stimuli across the palm in both the first (*p* < 0.001) and last training blocks (*p* = 0.006; **Figure 6A**). The Wilcoxon signed-rank test of Hypothesis 2 identified a significant improvement in the percentage of stimuli correctly interpreted on the first attempt between the first and the last training block (*p* < 0.001; **Figure 6B**). These improvements in the ability to identify stimuli during the training phase support the findings of the test-set analyses.

#### Detection of Stimulus Direction and Amplitude in Isolation

As in the test phase, we repeated the analysis considering the ability to correctly identify—in isolation—the direction and amplitude of the stimuli. For stimulus direction, we found a significant improvement in detection accuracy across all targets (*p* = 0.008). By contrast, we only found significant improvement in detection accuracy for the larger stimuli amplitudes (*p* = 0.004), but not for the inner-target stimuli (*p* = 0.888) or for all targets considered together (*p* = 0.072).

#### Nearest-Neighbor Analysis

As in the analysis of test-block performance, the value of the nearest-neighbor parameter in the training set was high for every subject in each training block (i.e., over 70% in each block). There was not a statistically significant improvement of this parameter between the first and last blocks of the training phase (*p* = 0.363).

#### Number of Attempts

On the training data set, we also report the number of attempts required for each stimulus to be identified correctly (**Figure 6C**). In support of Hypothesis 1, Friedman test found a statistically significant difference in the identification of stimuli across the palm in both in the first (*p* < 0.001) and last training blocks (*p* = 0.001).

### Validation on Stroke Survivors

**FIGURE 7:**
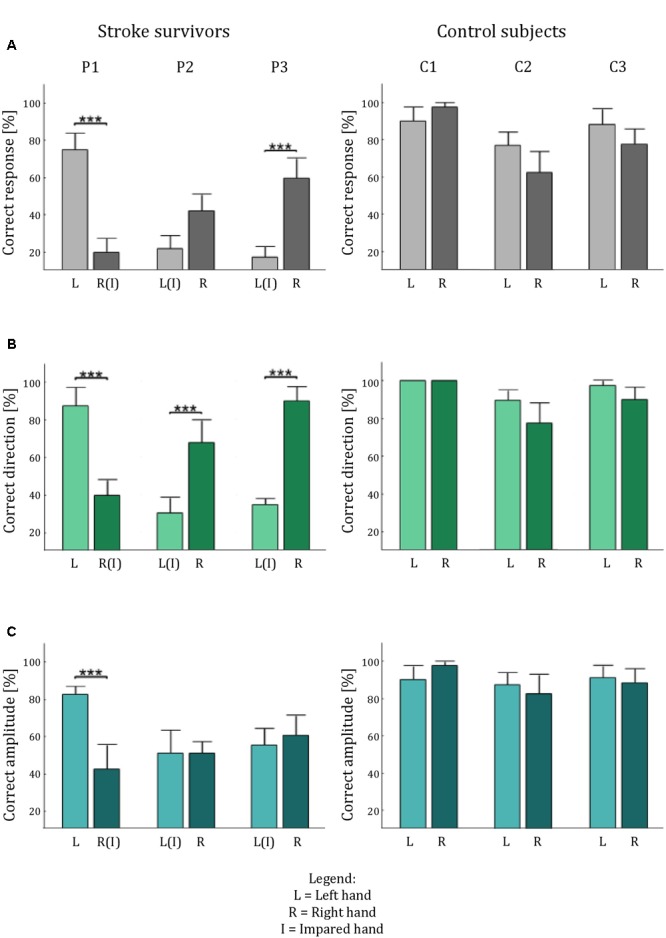
Tactile acuity in both hands of the stroke survivors and the control participants. The histograms show the average percentage performance in term of: correct answer **(A)**, correct direction **(B)**, and correct amplitude **(C)** for each hand for each subject. Error bars indicate the standard error of the mean. ^∗∗∗^*p* < 0.001.

The data of stroke survivors provide a first proof-of-concept assessment of *Tactile-STAR’s* ability to identify somatosensory deficits. Specifically, we investigated the ability of the participant to discriminate—in both hands—brush stimuli of two different amplitudes in each of the four cardinal directions. Given the heterogeneity of sensorimotor impairments expressed in stroke survivors, we used a single-subject analysis approach to probe for statistical differences of tactile perception between the two hands on a subject-by-subject basis. We expected to find significant differences between the two hands for each of the stroke survivors, but not for their matched, healthy, controls (**Figure 7**).

**FIGURE 8:**
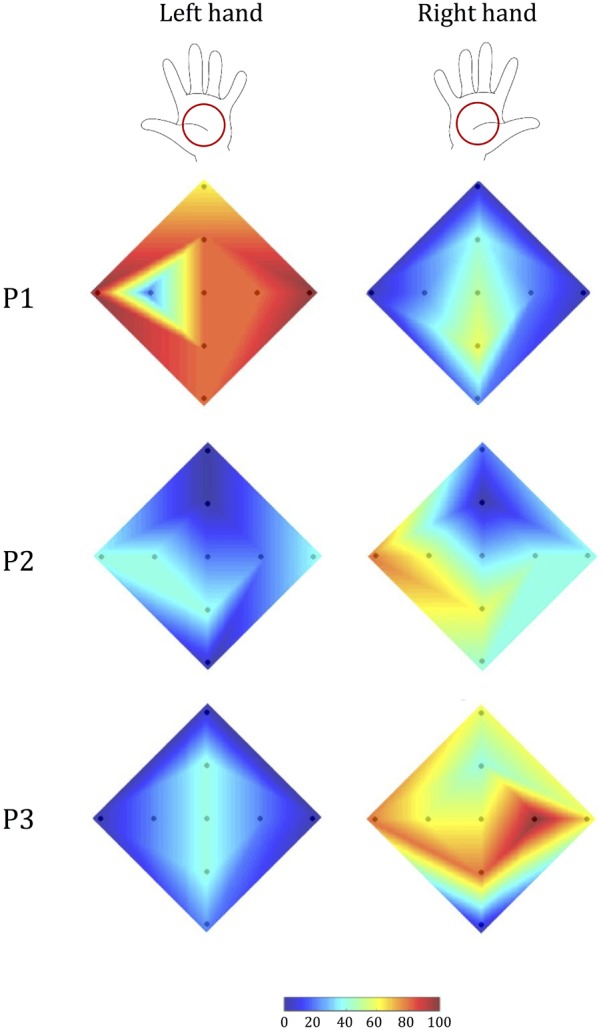
Tactile acuity in the left and right hand of stroke survivors. Colormap (as in **Figure 5**) of the percentage correct responses of each participant.

Stroke survivor P1 had a left-hemisphere lesion (left basal ganglia, internal capsule, and parietal lobe), which resulted in sensorimotor impairment on the right side of his/her body. Thus, we expected his/her ability to recognize tactile stimuli to be lower with the right hand than with the left (**Figure 8**). The experimental data confirmed this hypothesis: stimulus detection was worse with the right hand than with the left for all parameters analyzed (*p* < 0.001). By contrast, when we performed the same analyses with an age- and sex-matched control subject, we found no statistically significant differences in tactile perception between the two hands (*p* > 0.24 for all indicators).

Stroke survivor P2 had left-sided sensorimotor impairment (with a brain lesion located primarily in the right thalamus). Our experimental data showed that while the less-affected hand had better performance than the more-involved hand in terms of identifying the correct direction (*p* < 0.001), the participant expressed a bilateral difficulty in correctly identifying stimulus amplitude (*p* = 1), particularly in the upward direction (**Figure 8**). As expected, this participant’s control generally had markedly better performance, and did not show any significant difference between the two hands (*p* > 0.130 in all cases), although the performance was slightly better for the non-dominant hand.

Stroke survivor P3 had a right fronto-parietal, pre-Rolandic lesion (i.e., left-side impairment). As expected, he/she expressed greater difficulty in interpreting stimuli with the left hand than with the right hand (**Figure 8**) both in terms of overall correct response (*p* < 0.001) and in the identification of stimulus direction (*p* < 0.001). By contrast, the ability to discriminate between the two stimulus amplitudes was not significantly different between the two sides of the body, due to bilateral difficulty to correctly interpret the stimulus amplitude (*p* = 0.617). The control participant of this stroke survivor, instead, showed no significance difference for all the indicators we evaluated (all *p* > 0.24).

For each stroke survivor, the values of significance are reported without corrections for multiple comparisons; however, all the effects that were significant were robust to the Holm–Bonferroni corrections.

In summary, the *Tactile-STAR* device was able to identify specific differences in tactile acuity between the two hands in each of the three stroke survivors that participated in this study. These differences were due mainly to deficits in the ability to recognize the direction of tactile stimuli.

## Discussion

We developed and validated a new mechatronic system—the *Tactile-STAR*—for testing tactile acuity and treating somatosensory deficits in individuals with neurological diseases. Our preliminary validation testing supports the conclusions that: (1) The *Tactile-STAR* can characterize tactile perception and somatosensory deficits; and (2) A short bout of training with the *Tactile-STAR* system can improve the tactile perception of healthy individuals.

We obtained evidence in support of our first hypothesis in tests of *Tactile-STAR’s skin-brushing* stimulation mode. For each of 16 healthy participants, testing yielded a map of tactile perception on the hand. Results indicate that tactile acuity typically is non-uniform across multiple directions and distances from the center of the palm. This perceptual anisotropy may be the result of a non-uniform density of the mechanoreceptors in the palm of the hand (Kandel et al., 2000; Johansson and Flanagan, 2009) or the result of differences in the neural processing of the signals derived from those receptors (Kandel et al., 2000). Longo and Haggard (2011) found anisotropies of tactile size perception on the dorsum, but not on the palm of the hand. However, the task was different—the participants judged which of two tactile distances felt larger: the one aligned with the proximo-distal axis (along the hand), or the one aligned with the medio-lateral axis (across the hand). Future studies are needed to examine the utility of *Tactile-STAR* to characterize tactile perception with respect to skin-stretch displacement distance and direction, as well as to test the generalizability of target acquisition training under both skin stretch and skin brush modes on untrained movements guided by these tactile feedback signals.

In a small cohort of stroke survivors, we also performed a preliminary validation of the ability of the *Tactile-STAR* to detect sensory deficits after stroke. The device identified differences in tactile perception between the more- and less-impaired hands in each survivor. Intermanual differences were due mainly to impaired ability to recognize the direction of tactile stimuli in the more involved hand. Consistent with expectation, such differences were not found in control subjects, thus assuring that the pattern of results observed in the stroke survivors were not a result of handedness.

Taken together these results demonstrate that the *Tactile-STAR* system can offer quantitative and reliable measures of tactile acuity in the hand. We propose that the system also may be effective for characterizing tactile acuity in different dermatomes, and for monitoring changes due to aging, disease progression, or therapeutic intervention. In particular, we believe that the device could be used for testing different body parts, such as the feet, where deficits in the ability to detect sensory stimuli could be a sign of early onset of disease.

We obtained evidence in support of our second hypothesis in a test of short-term perceptual training with the *Tactile-STAR*. A mere 30 min of training with the system improved the ability of participants to recognize (and reproduce with one hand) specific *skin-brushing* stimuli applied to the other hand: In the post-training tests, healthy participants improved the percentage of stimuli recognized with respect to pre-training tests. Improvements in performance were detected both in terms of direction discrimination and in the accuracy of reproduction of “larger” stimuli directed to the periphery of the palm (i.e., the outer targets). This finding corroborates historical (Ruch et al., 1938) and recent reports (Carey et al., 1993; Yekutiel and Guttman, 1993) that somatosensory training can reduce somatosensory deficits. In the current study, improvements were significant for trained targets but not for untrained targets. We speculate that this specificity of training may have been due to the short duration of training. Indeed, this is an important point to further investigate since this perceptual learning is rooted in the low-level cortex and several studies suggest that it can generalize to different locations, but within a somatotopic framework and with a tactile memory distributed differentially according to the stimulus type (Harris et al., 2001; Harrar et al., 2014). Future studies should examine the ability of extended training with *Tactile-STAR* to improve detection and reproduction performance for both trained and untrained stimuli of multiple magnitudes. In particular, the experimenters noticed that increasing the number of short training sessions seems to be more beneficial than having fewer, longer sessions, suggesting possible difficulties in attending to stimuli for a long time and the risk of over-stimulation.

Training-dependent improvements in the ability to recognize both trained and untrained tactile stimuli would suggest that the *Tactile-STAR* could be a promising technology for the rehabilitation of somatosensation. This potential as a therapeutic tool should be verified in future studies by investigating whether the improvement is present and for how long it can last in neurological patients. If proved effective, the *Tactile-STAR* system could be impactful because somatosensory deficits are frequent outcomes of cerebral lesions (Feigenson et al., 1977). Not only are sensory deficits limiting on their own, but they also strongly limit the possibility of motor function recovery (Van Buskirk and Webster, 1955; Kusoffsky et al., 1982; Smith et al., 1983; Zeman and Yiannikas, 1989). Despite this evidence, training methods, devices, and protocols addressing somatosensory deficits and their rehabilitation are still limited.

The preliminary results presented here suggest that *Tactile-STAR* can be used to deliver augmented or supplemental feedback of hand position in space to guide goal-directed reaching actions. We are currently evaluating the extent to which training with *Tactile-STAR* can improve goal-directed actions performed with the more involved arm after a stroke. In this line of research, it is important to verify the efficacy of various information encodings (e.g., hand position error vs. state feedback; cf., Krueger et al., 2017). Future tests will compare the ability of skin-brush and stretch stimulations to enhance both tactile acuity and the performance of goal-directed reaching with the contralateral hand.

## Conclusion

We have developed a modular device that can apply controlled tactile stimulations to the palm. With modifications to the stimulator’s aperture, the device could be used to test the tactile acuity of different body parts. By investigating the two hypotheses described above for validating the system, the current study helps fill the gap in the literature pertaining to somatosensory assessment and retraining. Our future studies will focus on further developing the device and on advancing our understanding of tactile acuity and its training. The preliminary results described here motivate experiments aimed at both understanding the psychophysics of the sensory processing, and identifying optimal ways to enhance sensory abilities. Developing a mechanistic understanding of tactile somatosensation is important for a variety of applications that involve artificial interfaces designed to enhance sensorimotor control in both impaired and healthy motor systems. Specific examples include: rehabilitation (Krueger et al., 2017); prosthetics (Akhtar et al., 2014; Battaglia et al., 2017); brain–computer interface (Sketch et al., 2015); and sensory substitution and augmentation (Schorr et al., 2013; Quek et al., 2014a). Thus, the findings presented in this work are the first step toward a more ambitious goal of providing sensitive and reliable instruments that are capable of assessing and training tactile perception, and are suitable for enhancing sensory feedback in a variety of applications.

## Author Contributions

All the authors contributed to the design of the device. GB, PG, IN, RS, and MC designed the experimental protocols. GB, GC, and MC realized the device. PG selected the subjects and conducted the clinical evaluations. GB and PG collected the data. GB, IN, RS, and MC analyzed the results. All the authors contributed to discussing the results and to writing the manuscript. All authors read and approved the final manuscript.

## Conflict of Interest Statement

The authors declare that the research was conducted in the absence of any commercial or financial relationships that could be construed as a potential conflict of interest.
