# Supplementary material for: Tactile-STAR: A Novel Tactile STimulator And Recorder System for Evaluating and Improving Tactile Perception
Source: Front Neurorobot. 2018 Apr 6;12:12. doi: 10.3389/fnbot.2018.00012 (PMC5897626; doi:10.3389/fnbot.2018.00012)
Supplement: Supplementary file 1 [file Presentation_1.pdf]

Supplementary Material

Tactile-STAR: A novel tactile STimulator And Recorder system for evaluating and improving tactile perception

Giulia Ballardini, **Giorgio Carlini, Psiche Giannoni, Robert A Scheidt, Ilana Nisky, Maura Casadio**[*]

Correspondence: Maura Casadio, maura.casadio@unige.it

1. Direct and inverse kinematics of the stimulator and recorder devices

The pantograph structure has two degrees of freedom. Here, we report the kinematics equations (Campion, Wang, and Hayward 2005) that we used for the direct (Figure S1A), and the inverse problem (Figure S1B).

The horizontal position of the end effector, P3=(x3, y3)^T is determined by the two angles θ_1, θ_5, and the dimensions of the links a_i, i=1,…, 5, through the following direct kinematics equations:

$$P_2 = (x_2, y_2)^T = [a_1 \cos(\theta_1), a_1 \sin(\theta_1)]^T$$

$$P_4 = (x_4, y_4)^T = [a_4 \cos(\theta_5) - a_5, a_4 \sin(\theta_5)]^T$$

$$\|P_2 - P_h\| = \frac{(a_2^2 - a_3^2 + \|P_2 - P_4\|^2)}{(2\|P_2 - P_4\|^2)}$$

$$P_h = (x_h, y_h)^T = P_2 + \frac{\|P_2 - P_h\|}{\|P_2 - P_4\|}(P_4 - P_2) \qquad \text{(Eq S1.)}$$

$$\|P_3 - P_h\| = \sqrt{a_2^2 - \|P_2 - P_h\|^2}$$

$$P_3 = (x_3, y_3)^T = [x_h + \frac{\|P_3 - P_h\|}{\|P_2 - P_4\|}(y_4 - y_2), y_h - \frac{\|P_3 - P_h\|}{\|P_2 - P_4\|}(x_4 - x_2)]^T$$

where P_1 and P_5 (Figure S1) correspond to the positions of the two motors for the stimulator device, and the potentiometers for the recorder (a_5 is the distance between them and is fixed to be equal in the two devices). P_3 corresponds to the end effector of each device. Our devices use a symmetrical pantograph; this means that the length of a_1 is the same of a_4 and a_2=a_3 in both devices. The arm a_1 is called the proximal arm, and the arm a_2 is called the distal arm. The center of the reference frame (x, y) is fixed in P_1. Note that our equation P3 ignores for both x_3 and y_3 the redundant non-feasible solution.

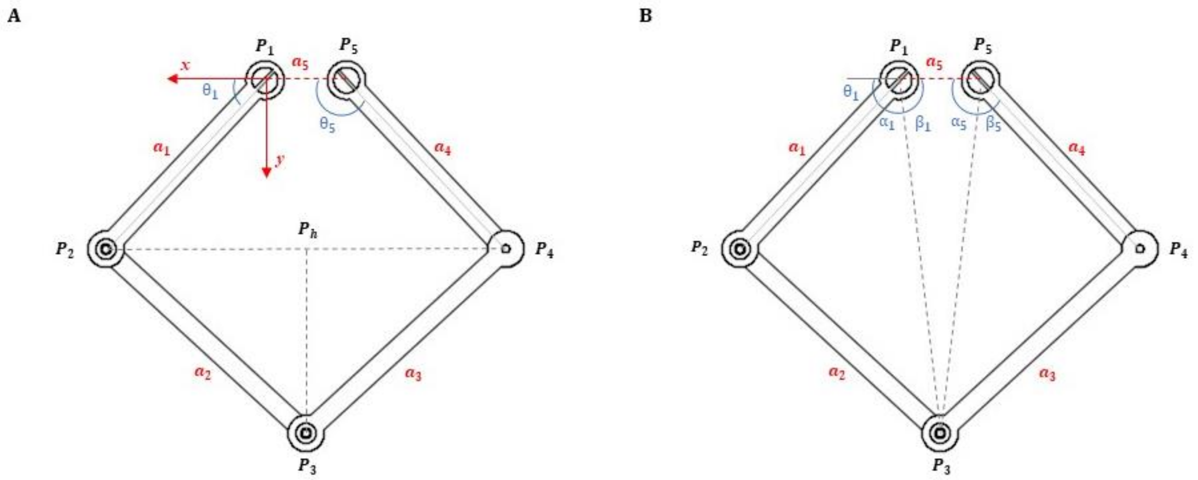

Figure S1. Model of kinematics of the pantograph structure used for the direct and the inverse problem of the kinematics

The inverse kinematics equations are used to find the angles θ_1 and θ_5 from the spatial coordinates x_3, y_3 of P_3. Specifically, to solve the inverse kinematic problem the pantograph is divided into three triangles as in Figure S1 Panel B. The angle α_1 is the angle of the triangle composed by the arms a_1 and a_2. The angles β_1 and α_5 are the angles of the middle triangle formed in the pentagon. The angle β_5 is the angle of the triangle made by the arm a_4 and a_3.

$$\theta_1 = \pi - \alpha_1 - \beta_1$$

$$\alpha_1 = \arccos\left(\frac{a_1^2 - a_2^2 + \|P_1, P_3\|}{2a_1\sqrt{\|P_1, P_3\|}}\right)$$

$$\beta_1 = \text{arctan2}(y_3, -x_3) \qquad \text{(Eq S2.)}$$

$$\theta_5 = \alpha_5 + \beta_5$$

$$\alpha_5 = \text{arctan2}(y_3, x_3 + a_5)$$

$$\beta_5 = \arccos\left(\frac{a_4^2 - a_3^2 + \|P_5, P_3\|}{2a_4\sqrt{\|P_5, P_3\|}}\right)$$

Reference:

Campion, G., Wang, Q., and Hayward, V. (2005). “The Pantograph Mk-II : A Haptic Instrument.” *IEEE/RSJ Int. Conf. Intelligent Robots and Systems*, 723–28.

2. Development of the device through 3D printers

All parts of the pantograph structure were manufactured by a Form2 stereolithographic printer (FormLabs Inc.) with a resolution of 0.05 mm. This printer polymerizes a liquid methacrylate photopolymer resin through a laser, with a spot size of 0.140 mm. We used Tough Resin to print the pantograph structure, and it was selected for its favorable mechanical properties, including Young's modulus and tensile strength. After polymerization, the printed part was washed twice with 90% isopropyl alcohol (IPA) for ten minutes each. IPA is a solvent that removes leftover uncured resin from the printed part. After the wash, the parts were placed under a 405 nm LED light for 120 minutes at 60°C. This second treatment increases the thermal and mechanical properties of the part (e.g., tensile strength, Young's modulus, flexural strength and modulus). Young's modulus before and after the treatment are 1.7 and 2.7 GPa, and the flexural strength at 5% strain is 20.8MPa and 60.6MPa, respectively. The remaining parts were manufactured by a DeltaWASP 2040 printer (WASP project). The DeltaWASP is a fused deposition model that works on an additive principle by laying down a filament of thermoplastic material (polylactic acid, PLA). This printer can print parts with lager dimension at lower cost, without need for additional processing after printing.

3. CAD model of the connection between twos links of the pantograph

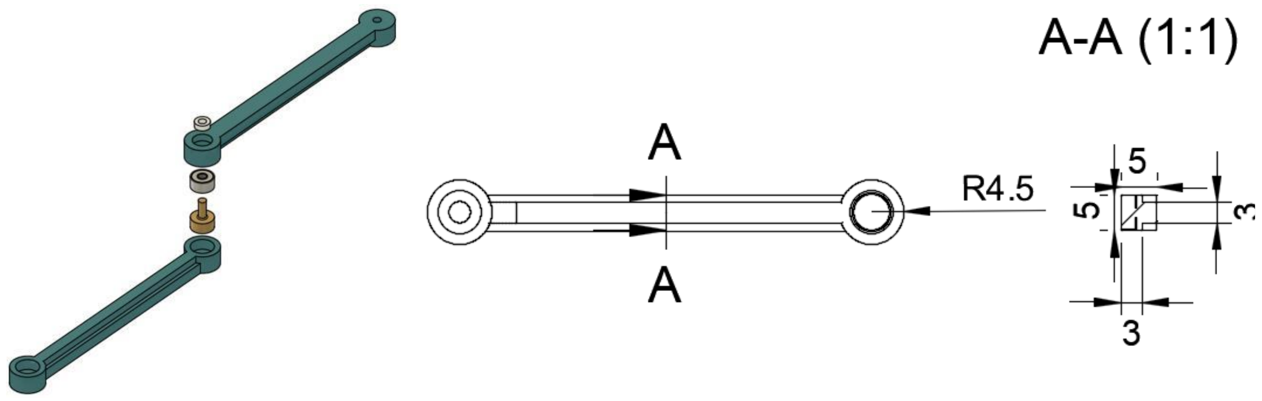

Figure S2. Left: CAD model of the connection between two links: a ball-bearing was fixed in one arm, and a brass axle was rigidly connected with the other arm. On the top of the axle is attached a plastic ring, which must maintain the connection fixed during the movement of the structure. Right: Drawing of a proximal arm of the pantograph structure (i.e., one of the arms attached to a motor) and its T-shaped cross-section (measurements are in mm).

4. Scheme of the electrical connections of the Tactile-STAR device

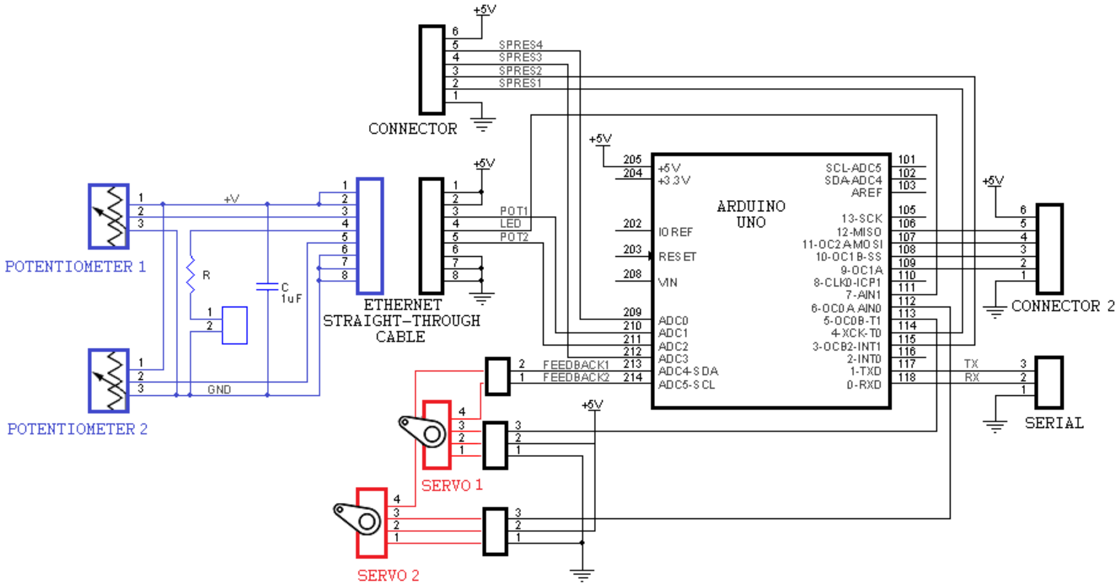

Figure S3. Scheme of the electrical connections of the Tactile-STAR device. The recorder and the stimulator are electrically connected to an Arduino Uno through two custom-printed circuit boards.
